# JBPOS0101 regulates amyloid beta, tau, and glial cells in an Alzheimer’s disease model

**DOI:** 10.1371/journal.pone.0237153

**Published:** 2020-08-13

**Authors:** Jihoon Jeong, Hyung Joon Park, Bo-Ram Mun, Ju Kyong Jang, Yong Moon Choi, Won-Seok Choi

**Affiliations:** 1 School of Biological Sciences and Technology, College of Natural Sciences, College of Medicine, Chonnam National University, Yongkang, Gwangju, Republic of Korea; 2 Bio-Pharm Solutions Co. Ltd, Suwon, Gyeonggi-Do, Republic of Korea; University of Florida, UNITED STATES

## Abstract

Alzheimer’s disease (AD) is the most prevalent neurodegenerative disease characterized by cognitive dysfunction and memory loss as the main symptoms. The deposition of amyloid beta (Aβ) and tau hyperphosphorylation are hallmarks of AD and are major therapeutic targets. However, the exact etiology has not yet been fully elucidated; thus, no drug that cures the disease has been approved. JBPOS0101 is a phenyl carbamate compound that has been tested as a drug for epileptic diseases. In our previous study, we showed that JBPOS0101 attenuated the accumulation of Aβ as well as the deficits in learning and memory in the 5xFAD mouse model. Here, we tested the dose effect (70 or 35 mg/kg) of JBPOS0101 on the memory defect and pathological markers and further investigated the underlying mechanisms in 5xFAD mice. In the behavior tests, JBPOS0101 treatment ameliorated deficits in learning and memory. Moreover, JBPOS0101 attenuated Aβ accumulation and tau phosphorylation. The elevated phosphorylation levels of the active GSK3β form (GSK3β-y216) in 5xFAD, which are responsible for tau phosphorylation, decreased in the JBPOS0101-treated groups. Furthermore, the elevation of reactive astrocytes and microglia in 5xFAD mice was attenuated in JBPOS0101-treated groups. These data suggest that JBPOS0101 may be a new drug candidate to lessen amyloid- and tau-related pathology by regulating glial cells.

## Introduction

Alzheimer's disease (AD) is one of the most common age-related neurodegenerative disorders. AD is characterized by cognitive dysfunction and memory loss. Though the exact etiology of AD is not yet fully understood, the primary cause is thought to be the deposition of intracellular neurofibrillary tangles and extracellular senile plaques [1]. Senile plaques consist of aggregates of amyloid-β (Aβ) peptide and dystrophic neurites [2]. Aβ peptides are produced through the amyloidogenic pathway by cleavage of the amyloid precursor protein (APP). The most common forms are Aβ40 and Aβ42, which are easily aggregated with one another and are thought to be the main cause of pathology in AD [3]. Aβ plaques begin developing in the neocortex and extend to other regions of the brain during the progression of the disease [4]. The binding of aggregated Aβ oligomers to neuronal receptors or synapses may affect neuronal functions and cause complications, such as neurodegeneration and cognitive dysfunctions [5]. In addition, Aβ fibrils induce glial activation and inflammatory responses [6]. The activation of astrocytes and microglia can be observed in AD, primarily surrounding aggregated Aβ [7]. When activated, proinflammatory cytokines and toxic products such as reactive oxygen species (ROS) and proteases, are released [8]. These may cause neuronal defects [9].

Metabotropic glutamate receptors (mGluRs) belong to a class of G-protein coupled receptors. They form a family of eight subtypes (mGlu1 to mGlu8) and are widely expressed in glial cells, including microglia and astrocytes, as well as neurons [10]. In the glial cells, mGluRs are involved in various functions, including cell proliferation, cytokine release, and glutamate transporter activity [11, 12]. However, the expression and role of mGluRs in astrocytes and microglia have not yet been fully defined.

JBPOS0101 is a small molecule (MW 229.05, 1-(2-chlorophenyl)-1-(S)-hydroxy-2-(S)-carbamoyloxy-propane, C10H12CINO3, Bio-Pharm Solutions Co. Ltd., Korea) that has been studied for its antiepileptic activity and approved for clinical trials [13]. The safety of the compound has recently been verified in a clinical trial (phase 1) [13]. In our previous study, we demonstrated the antagonistic activity of JBPOS0101 on mGluRs [14]. Moreover, JBPOS0101 attenuated the accumulation of Aβ and rescued the deficits in learning and memory in 5xFAD mice. Therefore, an investigation into the effect of JBPOS0101 on glial cells in an AD model is needed to define the exact role of the drug in glia-mediated AD regulation.

Neurofibrillary tangles (NFTs) are formed by bundles of hyperphosphorylated tau proteins in an aggregated form. NFTs are primarily generated in the entorhinal region, then extend to the limbic system and neocortex [15]. The formation of NFTs depends on several posttranslational modifications of tau in AD, and the most well-analyzed posttranslational modification is hyperphosphorylation. Tau has more than 70 potential phosphorylation sites, some of which are abnormally phosphorylated during the progression of AD [16–18]. In the AD brain, phosphorylation sites of tau are associated with aggregation processes, including incomplete binding and microtubule destabilization, triggering the conversion of pre-tangles to NFTs [19, 20].

In this study, we verified the effect of JBPOS0101 on memory dysfunction and Aβ accumulation in a 5xFAD model. Furthermore, we investigated the new mechanism of JBPOS0101 for regulating tau and glial cells in an AD model.

## Materials & methods

### Animals

We used female wild type (WT) and AD model mice, 5xFAD, which express five familial AD mutations, three genes associated with human APP: K670 N/M671 L (Swedish mutation), I716 V (Florida mutation), and V717I (London mutation); and two genes related to human PSEN1: M146 L and L286 V FAD [21, 22]. The 5xFAD mice were generously obtained from Dr. Inhee Mook-Jung (Seoul National University). The mice were housed under a 12 h light/dark cycle at a temperature of ~20–22°C. All mice were separated into single cages with an appropriate amount of food and sterilized water. All experiments and animal care were approved by the Institutional Animal Care and Use Committee of Chonnam National University.

### JBPOS0101 administration

Mice were divided into four groups: WT/vehicle, 5xFAD/vehicle, 5xFAD/JBPOS0101 (35 mg/kg, Bio-Pharm Solutions, Korea), and 5xFAD/JBPOS0101 (70 mg/kg). Administration began when mice were five months old. Each group was injected once daily for 14 days (IP: 30% PEG400 (vehicle, Sigma, USA) with or without JBPOS0101).

### Behavior tests

#### Open field tests

General locomotor activity was tested in an open-top square arena box (40 × 40 × 40 cm) under low light conditions [23]. Mice were individually placed at the center of the arena and allowed to explore the arena box for 5 min in the training phase, and 20 min in the test phase (WT: vehicle, n = 20, 5xFAD: vehicle, n = 12, JBPOS0101 35 mg/kg, n = 8, JBPOS0101 70 mg/kg, n = 8). At the end of each phase, the surface of the arena was cleaned with 1% acetic acid and 70% EtOH. The movement of the mice was recorded, tracked, and analyzed with an Any-maze system equipped with a digital camera (Stoelting, USA).

#### Morris water maze test

The Morris water maze test was performed to measure spatial learning and memory of the mice. An open circular pool (114 cm in diameter) filled with opaque water was used in the test [24]. Non-toxic paint was used to make the water opaque, and the water temperature was set to 24 ± 2°C. The platform (17 × 10.5 cm) was placed in one of the target quadrants and hidden below the water surface [25]. The test was undertaken in two phases, the acquisition phase and the probe phase. In the acquisition phase, mice were trained for four trials a day for four consecutive days (a total of 16 trials, WT: vehicle, n = 20, 5xFAD: vehicle, n = 12, JBPOS0101 35 mg/kg, n = 8, JBPOS0101 70 mg/kg, n = 8) [23]. In each trial, mice were allotted 60 s to swim, and when they arrived at the platform, the trial ended. When completed, the mice were left on the platform for 10 s. After completion (four days of acquisition phases), the probe test was conducted without the platform for 90 s. The time spent in the target quadrant and latency to the platform were measured with Any-maze software.

#### Y-maze

The Y-maze test was performed in a symmetrical acrylic Y-maze that consisted of three arms (each arm: 40 × 5 × 13 cm) separated by 120° angles. Mice were placed in the center of the maze and allowed to explore freely for 8 min (WT: vehicle, n = 20, 5xFAD: vehicle, n = 12, JBPOS0101 35 mg/kg, n = 8, JBPOS0101 70 mg/kg, n = 8). Arms were named in alphabetical order, and the exploration behavior of the mice was recorded using the camera attached to the ceiling. From the video, we counted the total number of arms entered manually and calculated the percentage of alternations. Percentage alternation was calculated to assess spatial memory: actual alternation/(possible alternation (total number of arm entries) -2) × 100 (%) [26].

#### Cross maze

Cross maze testing was conducted in a maze with four arms arranged at 90° angles. Mice began their exploration in the center of the maze (WT: vehicle, n = 20, 5xFAD: vehicle, n = 12, JBPOS0101 35 mg/kg, n = 8, JBPOS0101 70 mg/kg, n = 8). The exploration duration was 10 min. The alternative ratio was calculated similar to the percentage of spontaneous alternation in the Y-maze: actual alternation /(possible alternation (total number of arm entries) -3) × 100 (%).

#### Fear conditioning test

Conditioning and testing were conducted in a chamber consisting of two rooms (dark and light). The test was conducted for two days. On the first day, the mice were placed in a dark chamber and the tone (3 KHz, 75 dB, 30 s) was played to each mouse (WT: vehicle, n = 18, 5xFAD: vehicle, n = 12, JBPOS0101 35 mg/kg, n = 9, JBPOS0101 70 mg/kg, n = 4). When the tone ended, an electric foot shock (2 s, 1 mA) was delivered to the mice through a stainless steel grid floor. On the second day, mice were placed in the same chamber (dark) for 3 min without shocks or tones to measure freezing time related to contextual memory [27]. Freezing was defined as the complete absence of motion, including motion of the vibrissae, for a minimum of 2 s.

### Brain tissue preparation and immunohistochemistry

Mice were anesthetized with sevoflurane and perfused with PBS (phosphate buffered saline). After perfusion, the left hemisphere of the mouse brains was dissected and the right hemisphere brain was fixed with 4% PFA for one day [28]. The frozen brains were sectioned in a coronal manner (30 μm thick) using a cryostat (Leica CM3000, Leica Microsystems, Germany). Sections were stored in a deep freezer (-70°C) until immunohistochemical analysis. In all immunofluorescence labeling procedures, we rinsed off the brain sections and blocked the tissues with cold PBST (0.1% Triton X-100 in PBS) containing 5% BSA and 5% goat serum at room temperature. We incubated them with primary antibodies, including mouse anti-6E10 antibody (1:3000, Covance, USA), rabbit anti-GFAP antibody (1:4000, Dako, Germany), and rabbit anti- Iba1 antibody (1:3000, Wako, USA), diluted in PBS. After the first incubation step, the brain sections were washed thrice with PBS for 10 min each, and incubation with secondary antibodies followed. For secondary antibodies, goat Alexa Fluor® 488 conjugated anti-rabbit antibody and goat Alexa Fluor® 568 anti-mouse antibody (Invitrogen, USA) were prepared at a dilution of 1:2000 in PBS.

### Quantification and analysis of images

All images were taken using a fluorescence microscope (Leica DM LB2). Quantification and analysis were conducted using ImageJ software (NIH). The immunoreactivity of 6E10 was quantified as the percentage of immunoreactive cells per mm^2^ of tissue (%) or the staining intensity. At least three brain sections were analyzed from each animal. For the quantification of astrocytes and microglia, we determined the relative immunoreactivity from the stained slides. The intensity of GFAP and Iba-1 was measured using ImageJ software.

### Western blotting

Brain cortex was harvested in solution (50 mM Tris-HCl (pH 7.6), 0.01% Nonidet P-40, 150 mM NaCl, 2 mM EDTA, 0.1% SDS, 1 mM PMSF, and protease inhibitors). The lysates were mechanically homogenized (10 repeats) through a 20-gauge needle. Samples were centrifuged at 3000 rpm for 5 min at 4°C. Cytoplasmic proteins were harvested from mechanically dissociated pellets with a micro pipette, added to TNT buffer (50 mM Tris-HCl (pH 7.6), 150 mM NaCl, 0.1% Triton-X 100), and centrifuged at 13,000 rpm for 90 min at 4°C [29]. Another half of the hemi-brain cortex was homogenized with RIPA buffer containing 1% Triton X 100, 0.5% deoxycholic acid, 0.2% SDS, 150 mM NaCl, 2 mM EDTA, and protease inhibitors (PMSF, aprotinin, NaF, and Na^3^VO_4_ from Sigma). The homogenate was centrifuged at 13000 rpm for 30 min, and the supernatant was used [2]. Proteins were separated by SDS-PAGE with 10% or 15% SDS gels and transferred onto PVDF membranes (Immobilon P membrane, Milipore, USA). Membranes were then blocked in 5% skim milk in TBST (Tris-buffered saline-Tween®20) and immunolabeled with primary antibodies: 6E10 (1–16 of human Aβ) mouse monoclonal antibody (Covance), GSK3β-S9 (1:3000) rabbit monoclonal antibody, GSK3β-Y216 (1:3000) mouse monoclonal antibody, total GSK3β (1:3000) rabbit monoclonal antibody, PHF-1(1:3000) mouse monoclonal antibody, tau5 (1:2000) mouse monoclonal antibody (Abcam, USA), GFAP (1:4000) rabbit polyclonal antibody (Dako), and Iba1 (1:4000) rabbit polyclonal antibody (Wako). Blots were developed with a chemiluminescence reagent (AbFrontier, Korea) and quantified using ImageJ software.

### Statistical analysis

Behavioral data, images, and western blot data were analyzed using one-way ANOVA and post hoc analysis using Tukey’s post hoc test. All data were presented as means ± SEM. All p values < 0.05 were considered statistically significant.

## Results

### Locomotion and anxiety level were not affected by JBPOS0101 treatment in 5xFAD mice

**Fig 1:**
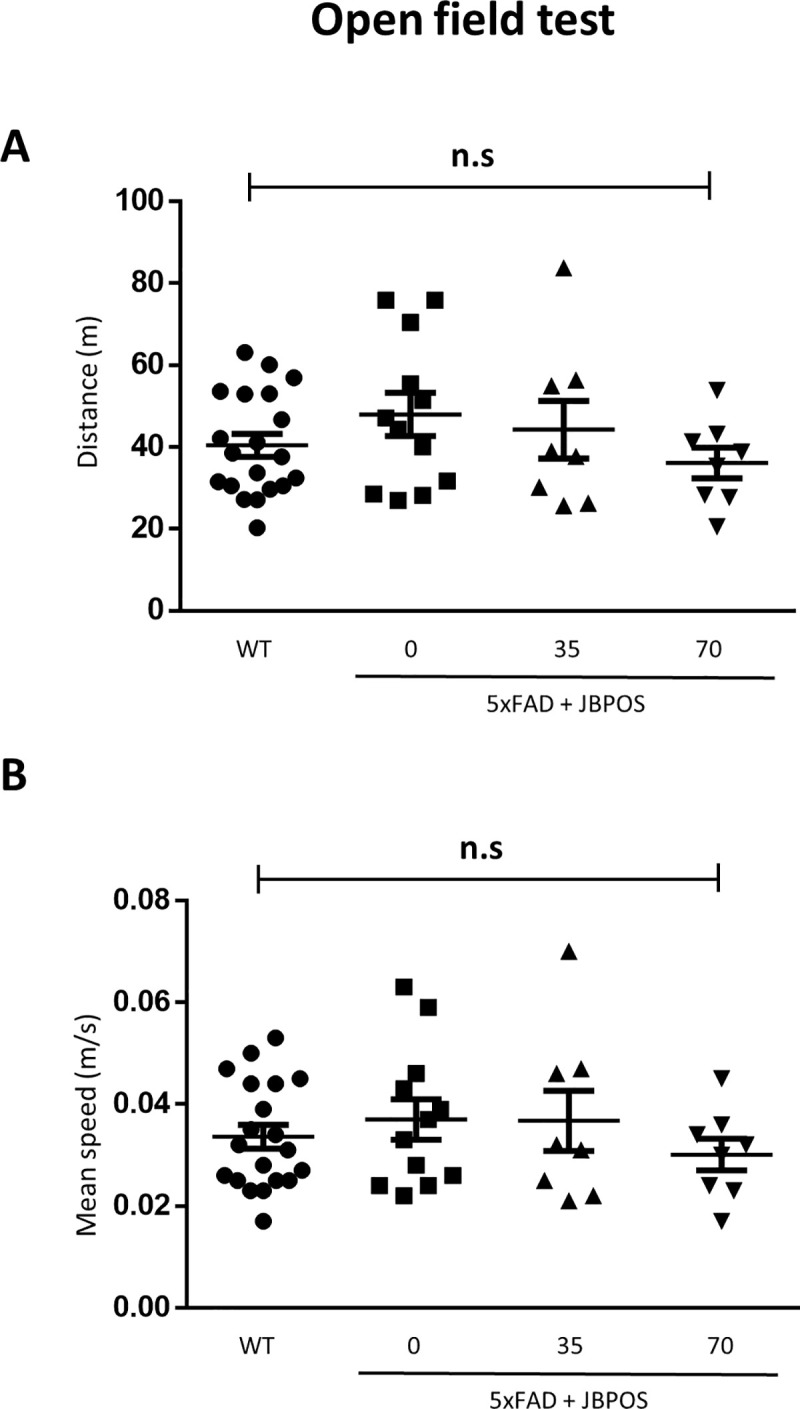
Locomotion was maintained normally by JBPOS0101 administration. Total distance (A) and mean speed (B) during exploration in the open-field arena (WT n = 20, 5xFAD vehicle n = 12, 5xFAD/JBPOS0101(35 mg/kg) n = 8, 5xFAD/JBPOS0101(70 mg/kg) n = 8). All the data show the mean value and error bars representing the standard error of the mean (SEM). WT, wild type. ns, not significant.

We used 5xFAD mice to investigate the effect of high (70 mg/kg) and low (35 mg/kg) doses of JBPOS0101 in an AD model. To test the effect of this compound on basal movement, locomotion was assayed with an open field test (OFT). No difference in basal locomotion among groups was identified in the OFT, including total distance and mean speed (Fig 1A and 1B). These results suggest that basal motor behavior was not affected by JBPOS0101 treatment.

### JBPOS0101 ameliorated memory loss in 5xFAD

**Fig 2:**
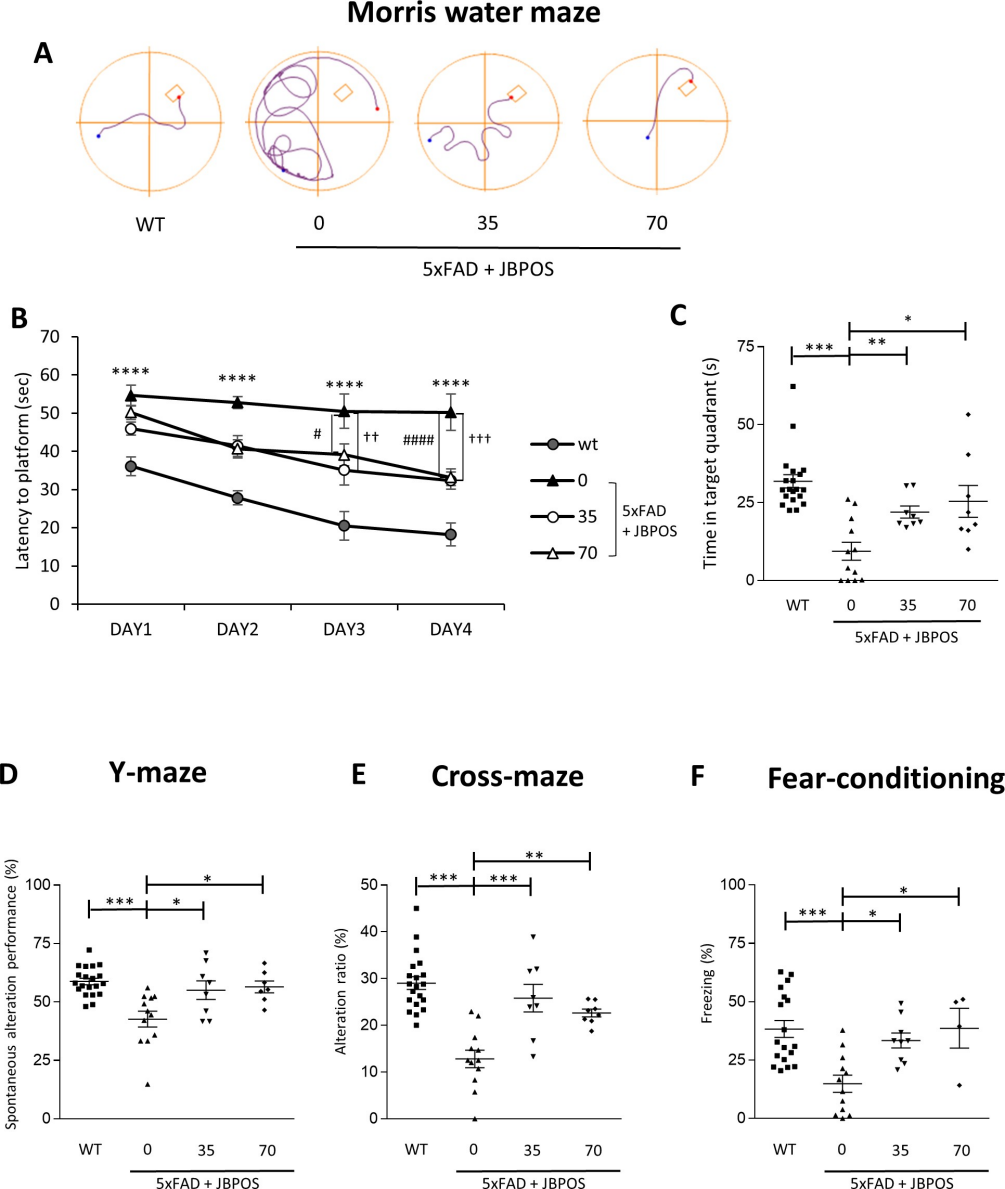
JBPOS0101 attenuated memory deficit in 5xFAD mice. (A) Representative track plots of WT, 5xFAD, and JBPOS0101-administered (35 mg/kg or 70 mg/kg) 5xFAD mice swimming during the last training trial of the Morris water maze test. (B) Latency to platform of the mice for four trials per day for four days (WT n = 20, 5xFAD vehicle n = 12, 5xFAD/JBPOS0101(35 mg/kg) n = 8, 5xFAD/JBPOS0101(70 mg/kg) n = 8). * indicates WT vs 5xFAD mice. ^#^ indicates 5xFAD/vehicle vs 5xFAD/JBPOS0101 (35 mg/kg). ^†^ indicates 5xFAD/vehicle vs 5xFAD/JBPOS0101 (70 mg/kg). (C) Time spent in the target quadrant was measured in the probe test (WT n = 20, 5xFAD vehicle n = 12, 5xFAD/JBPOS0101(35 mg/kg) n = 8, 5xFAD/JBPOS0101(70 mg/kg) n = 8). (D) Spontaneous alternation performance (the number of trials containing entries into all three arms per total number of entries into each arm-2) in the Y-maze (WT n = 20, 5xFAD vehicle n = 12, 5xFAD/JBPOS0101(35 mg/kg) n = 8, 5xFAD/JBPOS0101(70 mg/kg) n = 8). (E) Alternative ratio (%) of mice exploring the cross-maze. The alternative ratio was calculated similarly to that in the Y-maze test (WT n = 20, 5xFAD vehicle n = 12, 5xFAD/JBPOS0101(35 mg/kg) n = 8, 5xFAD/JBPOS0101(70 mg/kg) n = 8). (F) The response to stimulus in the fear-conditioning test was assessed through freezing time in the contextual memory test (WT n = 18, 5xFAD vehicle n = 12, 5xFAD/JBPOS0101(35 mg/kg) n = 9, 5xFAD/JBPOS0101(70 mg/kg) n = 4). All data are presented as mean and SEM. *p < 0.05, **p < 0.01, ***p < 0.005, ****p < 0.001, ^#^p < 0.05, ^##^p < 0.01, ^####^p < 0.001, ^†^p < 0.05, ^††^p < 0.01, ^†††^p < 0.005.

We performed a Morris water maze test to identify whether each dose of JBPOS0101 alleviates memory dysfunction, one of the main characteristics of AD. Over four days of training, the control or 5xFAD/JBPOS0101-treated groups exhibited a learning curve. However, the 5xFAD/vehicle group showed impairment in learning (Fig 2A and 2B). In the probe test, the time spent in the target quadrant significantly decreased in the 5xFAD/vehicle group, implying the impairment of location memory (Fig 2C). However, the JBPOS0101-administered 5xFAD/vehicle groups remained in the target quadrant much longer than the 5xFAD/vehicle group, suggesting that the memory deficit was attenuated by JBPOS0101 (Fig 2C). The effect of JBPOS0101 on short-term or working memory was examined in a Y-maze and cross maze. As shown in Fig 2D and 2E, alternation behavior significantly decreased in the 5xFAD/vehicle groups, indicating a memory defect in both tests. However, in JBPOS0101-treated groups, the memory defect was restored in the Y-maze and cross maze (Fig 2D and 2E).

The effect of JBPOS0101 on fear memory was tested by fear conditioning. The percentage of freezing time/total time was measured during the test. The percentage freezing time is shown in the graphs (Fig 2F), which indicate that fear memory was hampered in the 5xFAD/vehicle group, and significantly improved in the JBPOS0101-treated groups, confirming the protective effect of JBPOS0101 on the memory deficit.

These results suggest that JBPOS0101 may have a beneficial effect in ameliorating dysfunction in learning ability and memory in an AD model

### JBPOS0101 reduced Aβ deposition in the cortex and hippocampus regions of 5xFAD mice

**Fig 3:**
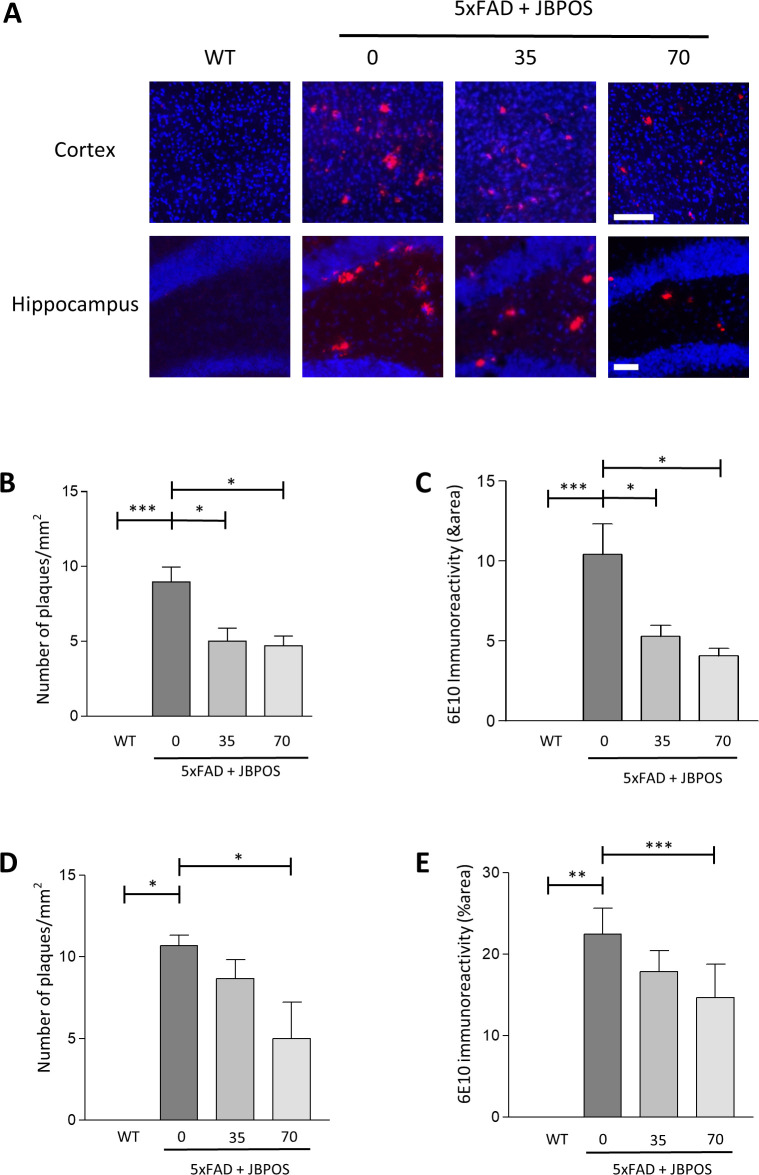
JBPOS0101 decreased Amyloid Beta (Aβ) aggregates in the cortex and hippocampal regions in 5xFAD mouse brains. (A) Representative images from the cortex and hippocampus regions of mouse brains. Brain slides were stained with 6E10 antibody (red) and DAPI (blue). Scale bars: 100 μm (cortex), 50 μm (hippocampus). (B, D) Quantitation of the number of amyloid plaques per mm^2^ in the cortex (B) and hippocampus (D). To count the number of plaques, three coronal sections of the similar level of cortex and hippocampus of each animal groups were used (WT n = 3, 5xFAD vehicle n = 3, 5xFAD/JBPOS0101(35 mg/kg) n = 3, 5xFAD/JBPOS0101(70 mg/kg) n = 3). (C, E) Quantification of 6E10 immunoreactivity in the cortex (C) and hippocampus (E). Immunoreactivity of 6E10 was measured using three sections from each brain containing cortex and hippocampus tissue (WT n = 3, 5xFAD vehicle n = 3, 5xFAD/JBPOS0101(35 mg/kg) n = 3, 5xFAD/JBPOS0101(70 mg/kg) n = 3). All data are presented as mean and SEM. *p < 0.05, **p < 0.01, ***p < 0.005.

Accumulation of Aβ has been implicated in memory dysfunction in AD. Therefore, we stained the cortex and hippocampus region of the brains with Aβ antibody to investigate whether JBPOS0101 has an effect on Aβ accumulation. Immunohistochemical staining showed that Aβ is accumulated in the cortex and hippocampus regions of 5xFAD mice (Fig 3A). However, JBPOS0101 reduced Aβ formation in 5xFAD mice (Fig 3A). In the quantification data, elevation of the number of plaques per mm^2^ and 6E10 immunoreactivity were significantly attenuated by JBPOS0101 in both the cortex and hippocampus regions of 5xFAD mice (Fig 3B–3E).

### Tau phosphorylation was prevented by JBPOS0101 administration in 5xFAD mice

**Fig 4:**
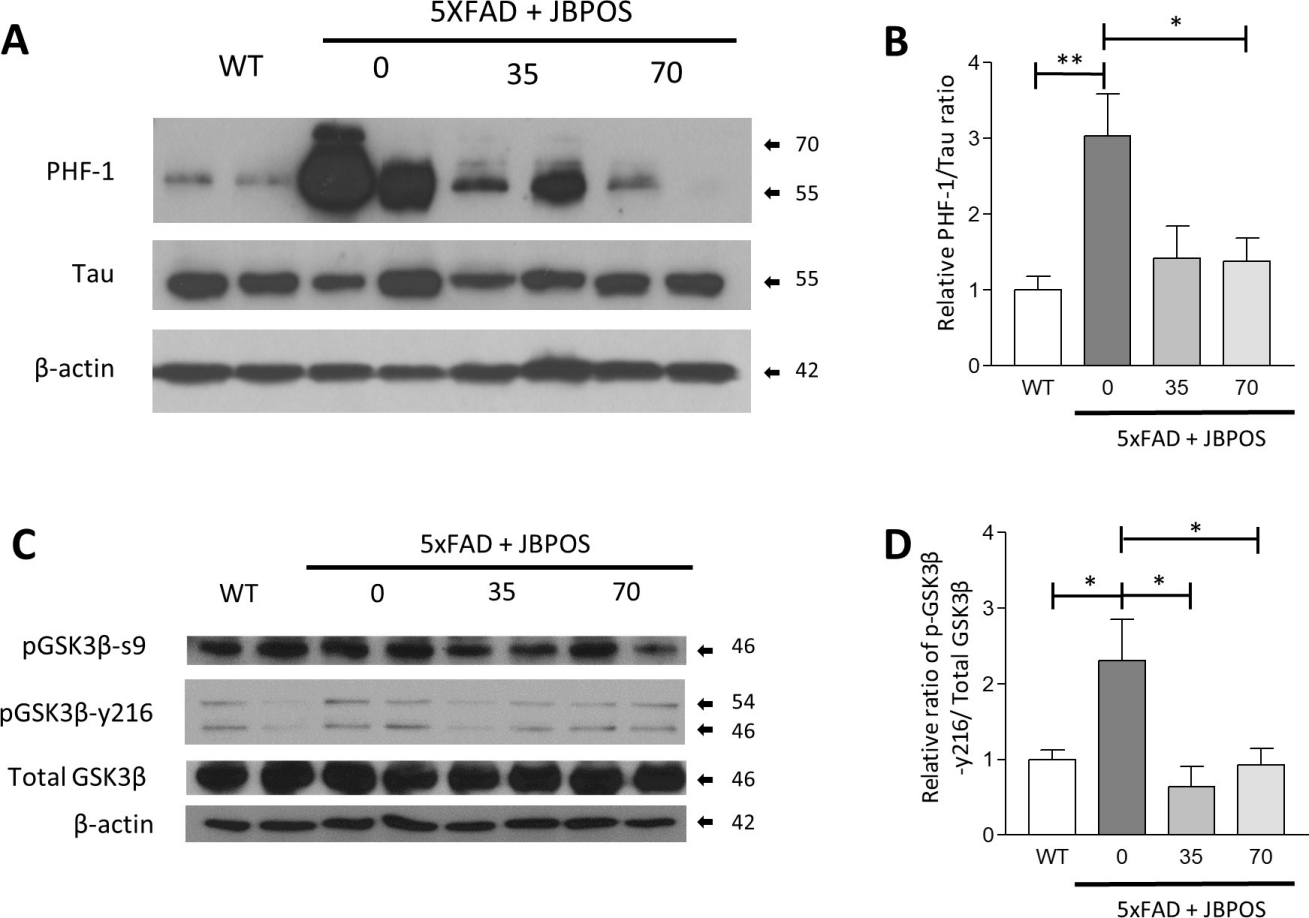
JBPOS0101 reduced tau phosphorylation in 5xFAD mice. Western blot of intracellular proteins. Protein samples were extracted from the cortex region of the brain with RIPA buffer. (A) Phosphorylated tau was analyzed using PHF-1 antibody (S1 Fig). (B) Quantification of the PHF-1 level relative to the Tau level (WT n = 5, 5xFAD vehicle n = 6, 5xFAD/JBPOS0101(35 mg/kg) n = 4, 5xFAD/JBPOS0101(70 mg/kg) n = 7). (C) Western blot analysis of GSK3β-S9, GSK3β-Y216, and total GSK3β (S2 Fig). (D) Quantitation of the relative ratio of GSK3β-Y216/β-actin (WT n = 6, 5xFAD vehicle n = 6, 5xFAD/JBPOS0101(35 mg/kg) n = 5, 5xFAD/JBPOS0101(70 mg/kg) n = 6). All data are presented as mean and SEM. *p < 0.05, **p < 0.01, ***p < 0.005.

The formation of NFTs is another pathological hallmark of AD and is caused by hyperphosphorylation of tau, a microtubule-associated protein. Conformational change and the accumulation of tau leads to the formation of paired helical filaments (PHF) [2]. We tested tau phosphorylation using anti-PHF1 antibodies, which detect tau phosphorylated at serine residues 396 and 404. Phosphorylation of tau protein was increased in 5xFAD mice and decreased in JBPOS0101-administered 5xFAD mice groups, compared to that in the 5xFAD/vehicle mice group (Fig 4A and 4B, S1 Fig).

To determine whether GSK3β is activated and contributes to tau phosphorylation, we attempted to detect the phosphorylation forms of GSK3β. Protein samples extracted from the brain cortex were analyzed by western blotting using antibodies specific for phospho-GSK3β (S9 or Y216) and total GSK3β. Phosphorylation in serine 9 of GSK3β and total GSK3β was largely unaltered (Fig 4C and 4D). On the contrary, phosphorylation levels of the active GSK3β form (GSK3β-y216) were elevated in 5xFAD compared to WT mice (Fig 4C and 4D). However, these were decreased in JBPOS0101-treated groups compared to the 5xFAD vehicle group (Fig 4C and 4D, S2 Fig).

These data suggest that JBPOS0101 might suppress tau phosphorylation by downregulating GSK3β active phosphorylation.

#### Activation of astrocytes and microglia in 5xFAD mice was ameliorated by JBPOS0101.

**Fig 5:**
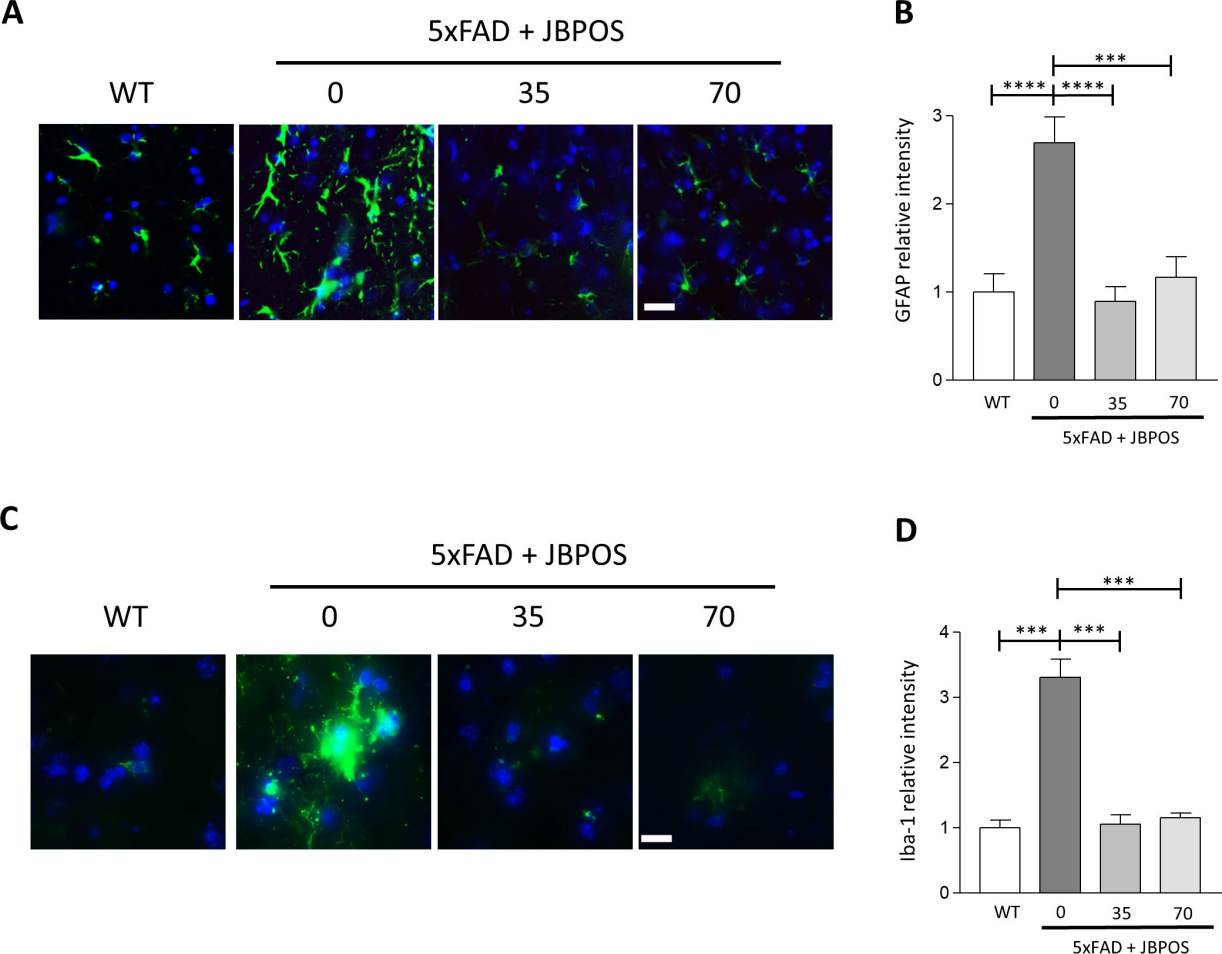
Activation of glial cells in 5xFAD mice was attenuated by JBPOS0101 administration. (A) Representative images of astrocytes from the hippocampus region of the brain. Brain sections were stained with GFAP antibody (green) and Hoechst33342 dye (blue). Scale bar: 50 μm. (B) Relative intensity of GFAP immunofluorescence (WT n = 5, 5xFAD vehicle n = 4, 5xFAD/JBPOS0101(35 mg/kg) n = 5, 5xFAD/JBPOS0101(70 mg/kg) n = 6). (C) Representative images of microglia in the hippocampus region of the brain stained with Iba1 antibody (green) and Hoechst33342 dye (blue). Scale bar: 50 μm (D) Quantification of Iba1 immunoreactivity (WT n = 4, 5xFAD vehicle n = 4, 5xFAD/JBPOS0101(35 mg/kg) n = 4, 5xFAD/JBPOS0101(70 mg/kg) n = 4). Two coronal sections from each mice brain tissue were used to verify GFAP and Iba1 intensity changes occur in hippocampus region. All data are presented as mean and SEM. ***p < 0.005, ****p < 0.001.

During AD progression, stimuli such as Aβ peptides activate astrocytes and microglia. To identify the role of JBPOS0101 in the activation of astrocytes and microglia in an AD model, the hippocampus region of the brains was stained with antibodies specific to the astrocyte maker, GFAP, and microglia marker, Iba1 (Fig 5). The number of reactive astrocytes was increased in 5xFAD mice, which was attenuated by JBPOS0101 administration (Fig 5A and 5B). Likewise, the number of Iba1-positive active microglia increased in 5xFAD mice (Fig 5C and 5D). JBPOS0101-treated 5xFAD mice had a smaller number of active microglia compared to the vehicle-treated mice, and the morphology of their microglia was similar to that in the resting state in WT mice (Fig 5C and 5D). The protein level of GFAP and Iba1 was also increased in 5xFAD mice, which was ameliorated by JBPOS0101 administration (S3 Fig).

These results suggest that JBPOS prevents the activation of astrocytes and microglia in 5xFAD mice to alleviate the resulting neurotoxicity and brain dysfunction, including memory defects.

## Discussion

5xFAD mice have been widely used as an AD model. These model mice exhibit amyloid deposition, memory loss, and cognition impairment. In this study, we confirmed pathological symptoms, including memory loss, Aβ accumulation, and tau phosphorylation, in 5xFAD mice. Previously, we reported that JBPOS0101 (35 mg/kg) improved brain function and attenuated Aβ accumulation in 5xFAD mice [14]. In the present study, we tested the effect of a high dose (70 mg/kg) of JBPOS0101 compared to a low dose (35 mg/kg). The high dose showed protective effects on memory defects and Aβ accumulation in 5xFAD mice. The high and low dose JBPOS0101 groups prevented memory loss in the Morris water maze. In addition, we found that the loss of fear memory was also attenuated by JBPOS0101 in 5xFAD mice. JBPOS0101 also had a beneficial effect on attenuating tau phosphorylation. The high and low dose JBPOS0101 treatment had effect on ameliorating Aβ accumulation and phosphorylation of tau protein in the 5xFAD model, again confirming the beneficial effects of JBPOS0101.

Tau phosphorylation can be mediated by several kinases [30]. In this study, the active form of GSK3β (GSK3β-y216) was increased in 5xFAD mice, which was attenuated by JBPOS0101. Kinase signals, including CDK5, can mediate the inhibition of GSK3β induced by the activation of mGluR. Therefore, exact effects of JBPOS0101 on cellular pathways related to AD pathology can be defined through further studies.

Previously, we had presented the antagonistic activity of JBPOS0101 on mGluRs. JBPOS0101 also affected astrocytes and microglia in this study. The morphology of those glial cells was close to that at the resting state when JBPOS0101 was administered, compared to the activated forms observed in the vehicle-treated 5xFAD group. In both astrocytes and microglia, conversion to the reactive form may be inhibited by JBPOS0101. In astrocytes, mGluR5 and mGluR3 can control the functions, including glutamate transporter activity and astrocyte-neuronal interactions [31]. In microglia, mGluRs regulate glutamate release, cell migration, and activation of the reactive microglia [32]. However, the expression and role of mGluRs are not fully defined in AD.

Overall, the main effect of the drug could be to reduce Aβ pathology. Thus, the reduction in the primary Aβ pathology would also lead to reductions in secondary pathologies including tau and glial cell modification. On the other hand, the prevention of astrocyte conversion to the reactive form and the resulting reduction in toxicity would attenuate microglial activation, or vice versa. Therefore, further investigation regarding the mechanisms of glial signaling in an AD model is needed to define the exact role of the drug in glia-related AD regulation.

## Supporting information

S1 Fig JBPOS0101 reduced tau phosphorylation in 5xFAD mice (A) The uncropped images of blots for Fig 4A. (B) The blots of other animals that were included in the quantitative data for Fig 4B (PHF-1, WT n = 5, 5xFAD vehicle n = 6, 5xFAD/JBPOS0101(35 mg/kg) n = 4, 5xFAD/JBPOS0101(70 mg/kg) n = 7).
(TIF)

S2 Fig JBPOS0101 reduced GSK3β-y216 phosphorylation in 5xFAD mice (A) The uncropped images of blots for Fig 4C. (B) The blots of other animals that were included in the quantitative data for Fig 4D (p-GSK3β-y216, WT n = 6, 5xFAD vehicle n = 6, 5xFAD/JBPOS0101(35 mg/kg) n = 5, 5xFAD/JBPOS0101(70 mg/kg) n = 6).
(TIF)

S3 Fig JBPOS0101 ameliorated glial cell activation. Western blot analysis of the protein sample extracted from cortex region with RIPA buffer. (A) GFAP was analyzed using anti-GFAP antibody (B) Iba-1 was analyzed using anti-Iba-1 antibody. (C, D) The uncropped images of blots for A and B (WT n = 2, 5xFAD vehicle n = 2, 5xFAD/JBPOS0101(35 mg/kg) n = 2, 5xFAD/JBPOS0101(70 mg/kg) n = 2).
(TIF)
