# Supplementary figures and images for: JBPOS0101 regulates amyloid beta, tau, and glial cells in an Alzheimer’s disease model
Source: PLoS One. 2020 Aug 13;15(8):e0237153. doi: 10.1371/journal.pone.0237153 (PMC7426148; doi:10.1371/journal.pone.0237153)

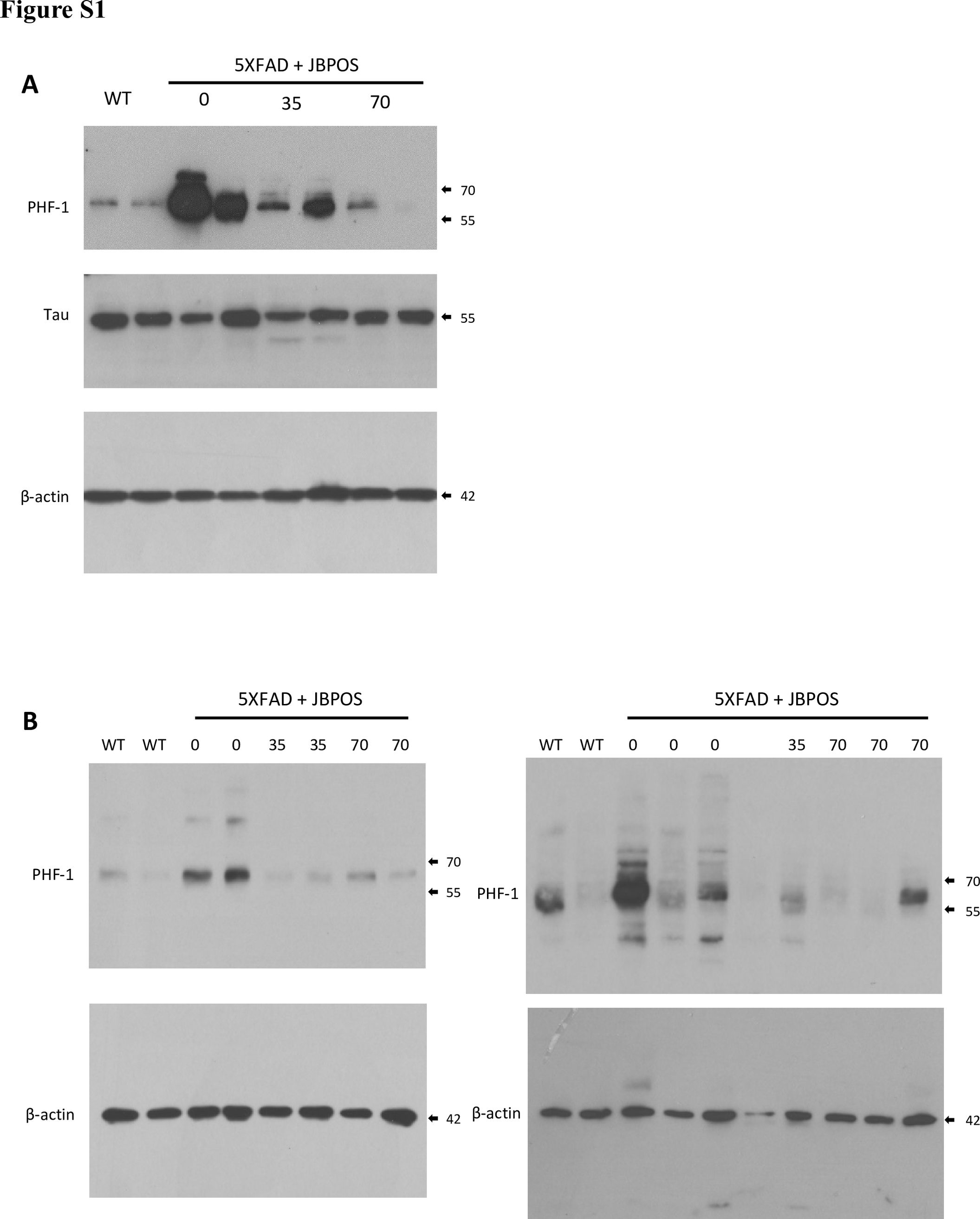

Supplement: S1 Fig — JBPOS0101 reduced tau phosphorylation in 5xFAD mice (A) The uncropped images of blots for Fig 4A. (B) The blots of other animals that were included in the quantitative data for Fig 4B (PHF-1, WT n = 5, 5xFAD vehicle n = 6, 5xFAD/JBPOS0101(35 mg/kg) n = 4, 5xFAD/JBPOS0101(70 mg/kg) n = 7). (TIF) [file pone.0237153.s001.tif]

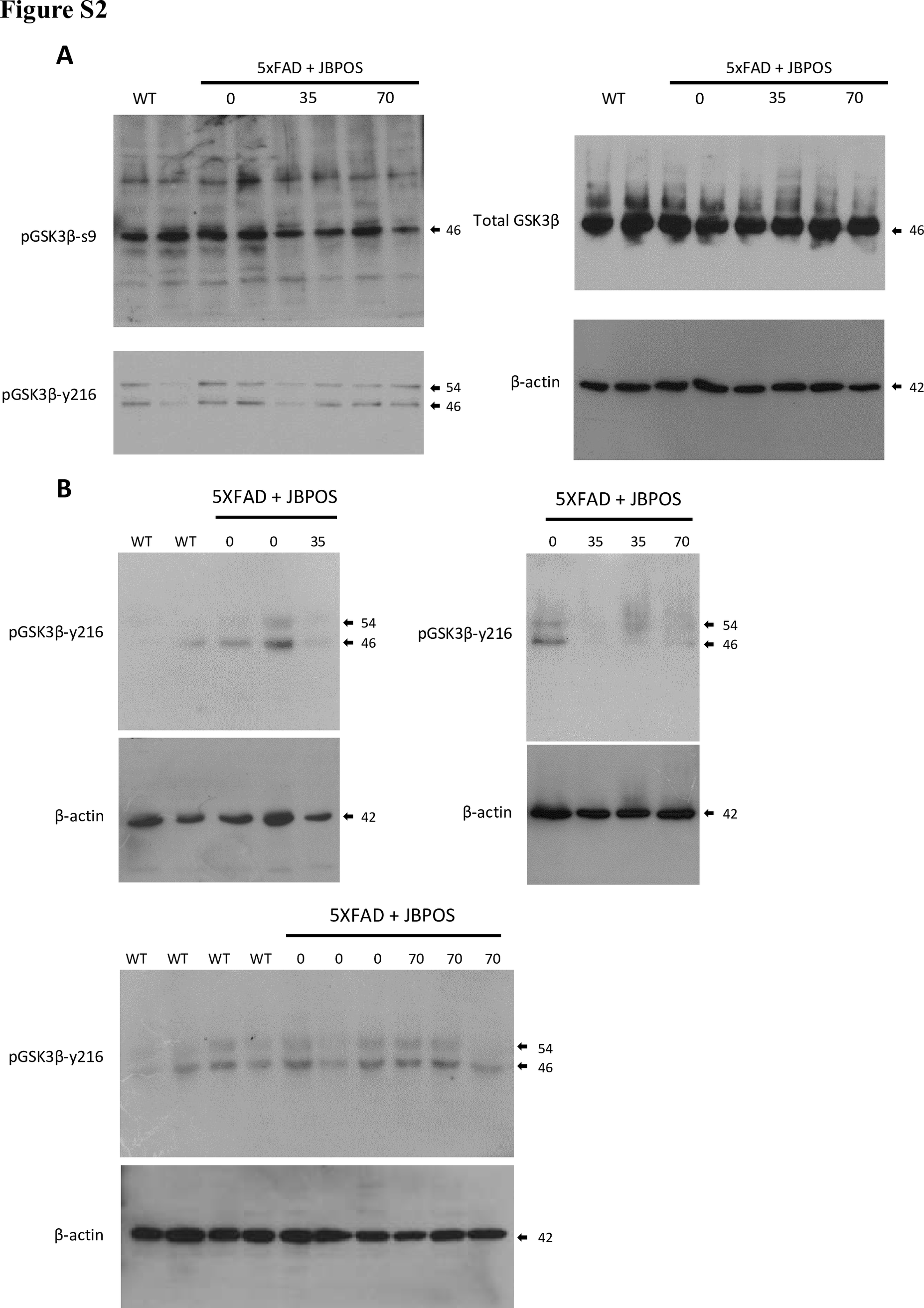

Supplement: S2 Fig — JBPOS0101 reduced GSK3β-y216 phosphorylation in 5xFAD mice (A) The uncropped images of blots for Fig 4C. (B) The blots of other animals that were included in the quantitative data for Fig 4D (p-GSK3β-y216, WT n = 6, 5xFAD vehicle n = 6, 5xFAD/JBPOS0101(35 mg/kg) n = 5, 5xFAD/JBPOS0101(70 mg/kg) n = 6). (TIF) [file pone.0237153.s002.tif]

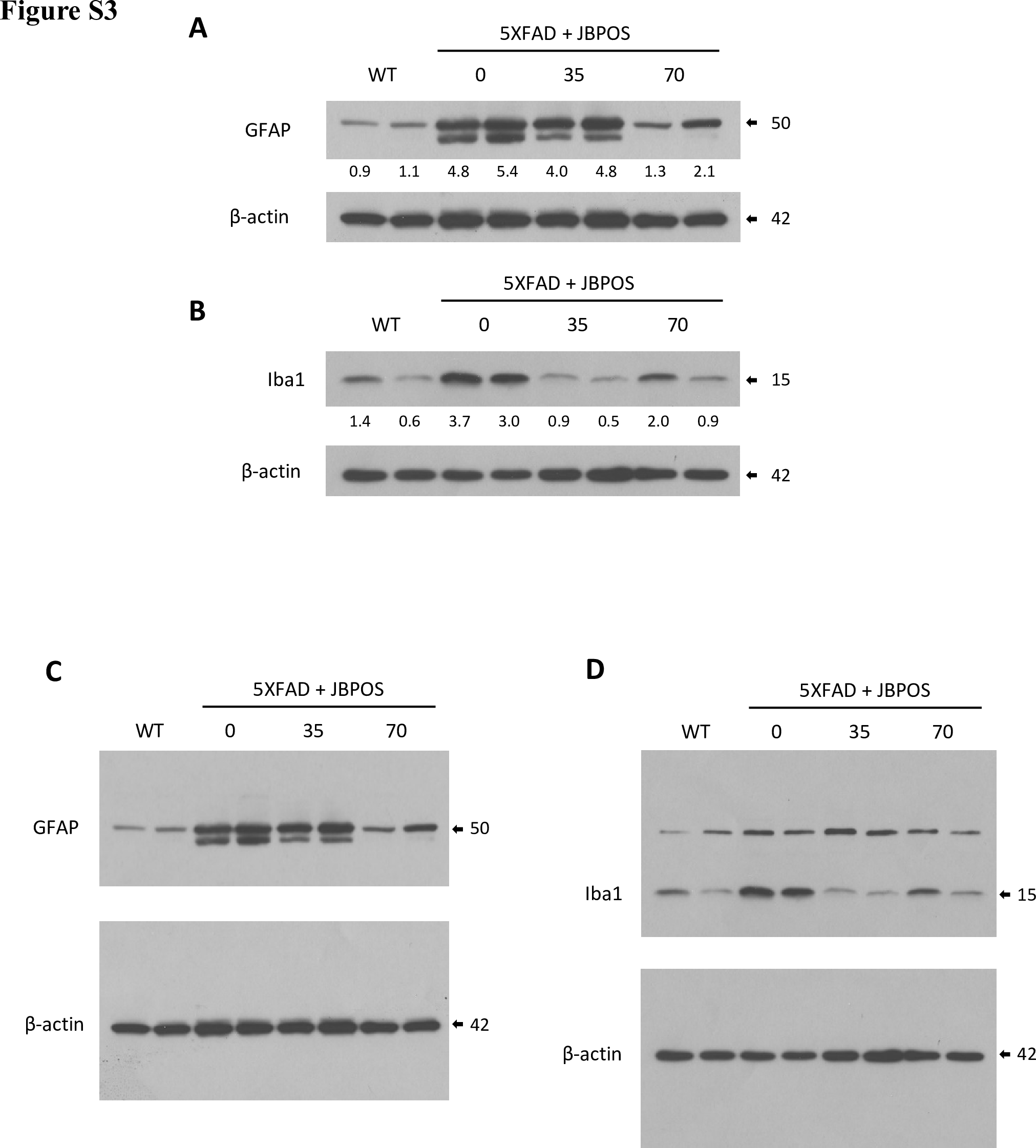

Supplement: S3 Fig — Western blot analysis of the protein sample extracted from cortex region with RIPA buffer. (A) GFAP was analyzed using anti-GFAP antibody (B) Iba-1 was analyzed using anti-Iba-1 antibody. (C, D) The uncropped images of blots for A and B (WT n = 2, 5xFAD vehicle n = 2, 5xFAD/JBPOS0101(35 mg/kg) n = 2, 5xFAD/JBPOS0101(70 mg/kg) n = 2). (TIF) [file pone.0237153.s003.tif]
